# Characterization of retinal nerve fiber layer thickness changes associated with Leber’s hereditary optic neuropathy by optical coherence tomography

**DOI:** 10.3892/etm.2013.1430

**Published:** 2013-11-27

**Authors:** YIXIN ZHANG, HOUBIN HUANG, SHIHUI WEI, HUAIYU QIU, YAN GONG, HONGYANG LI, YANLI DAI, ZHAOCAI JIANG, ZIHAO LIU

**Affiliations:** Department of Ophthalmology, Chinese PLA General Hospital, Beijing 100853, P.R. China

**Keywords:** Leber’s hereditary optic neuropathy, optical coherence tomography, retinal nerve fiber layer

## Abstract

In the present study, the changes in the retinal nerve fiber layer (RNFL) thickness associated with Leber’s hereditary optic neuropathy (LHON) were examined by Cirrus high definition-optical coherence tomography (OCT), and the correlation between the RNFL thickness and the best corrected visual acuity (BCVA) was evaluated. A cross-sectional study was performed. Sixty-eight eyes from patients with LHON and 30 eyes from healthy individuals were scanned. Affected eyes were divided into 5 groups according to disease duration: Group 1, ≤3 months; group 2, 4–6 months; group 3, 7–9 months; group 4, 10–12 months; and group 5, >12 months. The RNFL thickness of the temporal, superior, nasal and inferior quadrants and the 360° average were compared between the LHON groups and the control group. The eyes in groups 1 and 2 were observed to have a thicker RNFL in the superior, nasal and inferior quadrants and a higher 360°-average RNFL thickness compared with those of the control group (P<0.05), the RNFL was observed to be thinner in the temporal quadrant in groups 1 and 2. The eyes in groups 3 and 4 showed a thinner RNFL in the temporal (P=0.001), superior and inferior (both P<0.05) quadrants, and a lower 360°-average RNFL thickness as compared with controls (P=0.001). No significant correlation was identified between BCVA and RNFL thickness. RNFL thickness was observed to undergo a unique process from thickening to thinning in the patients with LHON. Changes in different quadrants occurred at different time periods and the BCVA was not found to be correlated with RNFL thickness.

## Introduction

Leber’s hereditary optic neuropathy (LHON) is a maternally inherited disease characterized by acute or subacute bilateral visual loss in young adulthood, particularly in males, with a median age of onset of 24 years (1,2). A study identified that >95% of LHON cases were caused by three point mutations of mitochondrial DNA (mtDNA): G11778A, T14484C and G3460A (3). However, the incomplete penetrance implicates that the mtDNA mutations are necessary but do not determine LHON, and additional genetic or environmental factors are required to trigger the pathological processes (4).

Histopathological descriptions of molecularly characterized patients with LHON have demonstrated a marked loss of retinal ganglion cells and their axons (5,6). The small-caliber fibers of the papillomacular bundle (PMB) are selectively lost at a very early stage of the pathological process, which eventually extends to the rest of the nerve, resulting in optic atrophy (7). According to disease duration, LHON may be divided into three stages: The preclinical stage, the acute/subacute stage (defined as ‘early’ within 6 months from onset; E-LHON) and the atrophic phase (>6 months; A-LHON), with 6-months being the mean time for the development of optic atrophy (8). A follow-up study showed evident optic atrophy and a stable visual acuity remaining at the lowest level after 6 months (9).

Optical coherence tomography (OCT) is a novel noninvasive, noncontact diagnostic technology, which is capable of performing high-resolution imaging of the transverse section of the retina *in vivo* and in real time (10). OCT has been used extensively to measure the retinal nerve fiber layer (RNFL) thickness and the macula lutea in patients with optic nerve and retinal diseases.

Since the RNFL thickness begins to change prior to disease onset, analyzing only the changes in RNFL thickness following disease onset may not be sufficient. Thus far, to the best of our knowledge, no previous studies have identified the changes of RNFL thickness that are associated with a cycling period in patients with LHON. In the present study, the changes in RNFL thickness in each quadrant were examined in patients with LHON at different disease durations, and the correlation between RNFL thickness and the best corrected visual acuity (BCVA) was investigated.

## Patients and methods

### Ethical considerations

This study was approved by the ethics committee of the Chinese PLA General Hospital (Beijing, China). The ethics committee approved the screening, inspection and data collection of these patients, and all patients provided written informed consent. All experiments followed the provisions of the Declaration of Helsinki.

### Patients

All patients with LHON diagnosed by mtDNA analysis in the Chinese PLA General Hospital (Haidian, China) between September 1, 2011 and March 31, 2013 were recruited. These patients were evaluated prospectively by ophthalmic tests, comprising BCVA, non-contact intraocular pressure measurements, slit-lamp microscopy, ophthalmoscopy and OCT. Patients were excluded according to the following criteria: Patients with retinal diseases and/or optic nerve diseases other than LHON; patients who were unable to accept OCT examination; patients with nystagmus whose OCT images were not stable; and patients with an OCT signal intensity of <6.

The recruited patients with LHON were divided into 5 diagnostic groups according to the duration of eye symptoms: Group 1, ≤3 months; group 2, 4–6 months; group 3, 7–9 months; group 4, 10–12 months; and group 5, >12 months.

Age- and gender-matched control individuals were recruited following the routine visual acuity testing of volunteers at the hospital. The control individuals underwent the same tests as those used to evaluate the patients with LHON. Based on OCT results, the eye with the better OCT signal was selected in each individual.

### OCT analysis

OCT scanning was performed by Cirrus high definition-OCT (software version 3.0, model 4000; Carl Zeiss Meditec, Inc. Dublin, CA, USA). Real-time image scans (27,000 A-scans/sec) were performed, an axial resolution of 5 microns was adopted and data were restructured as a 3-dimensional cube. RNFL thickness measurements were acquired using the optic disk cube 200×200 protocol and were analyzed the using optic nerve head (ONH) and RNFL oculus utro (OU) analysis protocols. BCVA examinations were performed using the logMAR visual testing chart (11).

All OCT scanning was performed in a darkroom by the same technician. Patients with a pupil diameter of <2 mm underwent mydriasis. In these patients, internal fixation was used whenever possible. If the patient was not able to see the internal fixation, they were asked to observe the external fixation using the fellow eye. If the method described above was infeasible for a patient, they were asked to move their eyes laterally during the scan acquisition until the image of the optic disc appeared on the screen of the operator. Each eye was rescanned until a good quality was obtained and an image was recorded for each eye. Statistical analyses were performed for the 360°-average RNFL thickness and the RNFL thickness in the temporal, superior, nasal and inferior quadrants.

### Statistical analysis

Statistical analysis was performed with SPSS software, version 19.0 (SPSS Inc., Chicago, IL, USA). Quantitative data were analyzed by the method of variance analysis with least significant difference multiple comparisons post hoc test. Linear correlation analysis was used for comparisons between the RNFL thickness and the BCVA. P<0.05 was considered to indicate a statistically significant difference.

## Results

### Demographic data of patients

**Figure 1:**
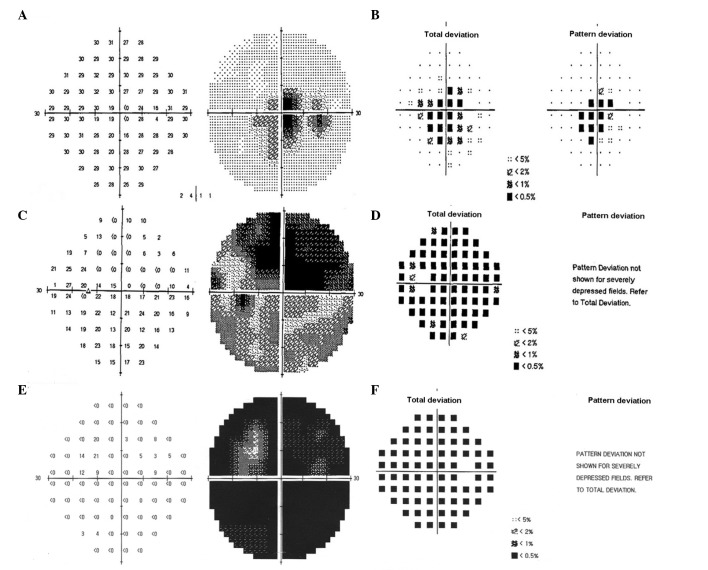
OCT scanning visual-field report of three typical patients. The central visual field defect degree aggravated gradually to diffuse defects in these three patients. (A) Patient 1 had a central visual field defect to a lesser degree; (B) 3-dimensional (3D) cube representation of results for patient 1 after processing. (C) Patient 2 showed a serious central visual field defect; (D) 3D cube representation of results for patient 2 after processing. (E) Patient 3 showed a more serious central visual field defect; (F) 3D cube representation of results for patient 3 after processing. OCT, optical coherence tomography.

A total of 68 eyes from patients with LHON (males, n=61; females, n=7) and 15 eyes from healthy individuals (males, n=10; females, n=5) were included. Table I presents demographic data of the study cohorts. Patients with a LHON duration of >12 months had a relatively older age and a longer mean duration of the disease; in this group, the longest duration of LHON was 3 years, but the LogMAR evaluation showed no statistically significant difference between disease groups. Fig. 1 demonstrates the OCT scanning visual-field report of three typical patients; the degree of the central visual field defect was aggravated gradually to diffuse defects in these three patients.

### RNFL thickness variation

**Figure 2:**
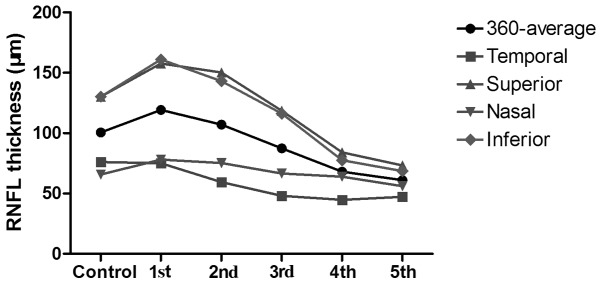
RNFL thickness in the temporal, superior, nasal and inferior quadrants, and the 360°-average in each group. RNFL, retinal nerve fiber layer; group 1, disease duration ≤3 months; group 2, disease duration 4–6 months; group 3, disease duration 7–9 months; group 4, disease duration 10–12 months; group 5, disease duration >12 months.

To compare RNFL thickness by OCT in patients with LHON and the control group, the changes in RNFL thickness were investigated at 3, 6, 9 and 12 months following onset. The mean RNFL thickness in each group is shown in Table II. The OCT scans show the RNFL to be temporarily relatively thicker in patients with LHON within 3 months from the time of disease onset. After 6 months, the 360°-average RNFL thickness and the RNFL in all quadrants (temporal, superior, nasal and inferior) became thinner and progressively thinned over 12 months. The changes in RNFL thickness in each quadrant and the 360° averages for the different time course groups are displayed in Fig. 2.

### Changes in the superior and inferior quadrant and 360°-average RNFL thickness

Compared with the control group value, the 360°-average RNFL thickness was significantly higher in group 1 (P=0.026), and lower in groups 3, 4 and 5 (P=0.005, <0.001 and <0.001, respectively). The 360°-average RNFL thickness in groups 3, 4 and 5 was significantly increased compared with those in group 1 (P=0.016, 0.001 and <0.001, respectively) and group 2 (P=0.006, <0.001 and <0.001, respectively). In groups 4 and 5, the 360°-average RNFL thickness was significantly increased compared with that of group 3 (P=0.002 and <0.001, respectively), while the RNFL thickness was not observed to be significantly different between groups 4 and 5.

The changes in RNFL thickness in the superior and inferior quadrants were comparable with those in the 360°-average RNFL thickness.

### Changes in RNFL thickness in the nasal quadrant

The RNFL thickness in the nasal quadrant was significantly increased in groups 1, 2 and 5 compared with that of the control group (P=0.046, 0.023 and 0.005, respectively). The RNFL thickness of the nasal quadrant was significantly reduced in groups 4 and 5 compared with that in group 1 (P=0.048 and 0.002, respectively), and in groups 3, 4 and 5 compared with that in group 2 (P=0.048, 0.019 and <0.001, respectively). The RNFL thickness of the nasal quadrant was significantly reduced in group 5 compared with those in group 3 (P=0.01) and group 4 (P=0.013).

### Changes in RNFL thickness in the temporal quadrant

The RNFL thickness of the temporal quadrant was significantly increased in groups 2, 3, 4 and 5 compared with that in the control group (P=0.005, <0.001, <0.001 and <0.001, respectively). The temporal RNFL thickness was significantly decreased in groups 3, 4 and 5 compared with that in group 1 (P=0.019, 0.002 and 0.002, respectively), and significantly decreased in groups 4 and 5 compared with that in group 2 (P=0.016 and 0.043, respectively). No other statistically significant differences were identified between the groups.

### Correlation between RNFL thickness and BCVA

In the present study, logMAR values were used as a measure of the BCVA in each group. Following analysis, the RNFL thickness in the four quadrants and the 360° average showed no linear correlation with BCVA (P>0.05; data not shown).

## Discussion

With the development and continuous upgrading of technology, OCT has become one of the most effective technologies with which to study optic nerve and retinal diseases (12,13), particularly regarding the anatomical structure of the retina and RNFL thickness. Recently, OCT has been commonly applied in optic neuropathy research, such as the study of glaucoma, optic neuritis and multiple sclerosis (14,15). However, studies of LHON are limited in the literature.

A previous study of LHON by OCT showed that unaffected carriers demonstrated thicker RNFL in the temporal and inferior quadrants than the control (16). The RNFL thickness in patients with E-LHON and A-LHON also differs. In a cross-sectional study, eyes with E-LHON showed a thicker RNFL in the temporal quadrant compared with that of the healthy control group, and no significant changes were detected in other quadrants, whereas eyes with A-LHON demonstrated a thinner RNFL in all measurements (17). Furthermore, a cohort study of four patients with molecularly defined LHON by Barboni *et al*(18), demonstrated that the temporal and inferior quadrants showed a statistically significant increase of RNFL thickness between the presymptomatic stage and disease onset. With the exception of the temporal quadrant, the RNFL thickness showed a statistically significant increase between the presymptomatic stage and the 3-month follow-up. A significant reduction of RNFL thickness was detected in all but the nasal quadrant between the presymptomatic stage and the 9 month follow-up (18).

A previous study showed that the RNFL thickness increased significantly in the temporal quadrant and marginally increased in the inferior quadrant of non-invasive carriers (16). Combined with the findings of the present study, this suggests that RNFL in the temporal and inferior quadrants thickens prior to the occurrence of the disease, but swelling in the temporal quadrant recovers gradually within 3 months following disease onset. In the pathological process, small-caliber fibers of the PMB are selectively lost at a very early stage and this loss is then extended to the other nerve fibers, resulting in diffused optic atrophy (7,19). As the RNFL in the temporal quadrant is mainly composed of the PMB, its thickness was altered earlier than that of the other quadrants. Between 4–6 months, RNFL in the temporal quadrant was significantly reduced, suggesting that the RNFL in the temporal quadrant started to atrophy and the swelling of RNFL in other quadrants began to subside. Between 7 and 9 months, the RNFL in the superior, nasal and inferior quadrants had started to shrink with the reduction in the superior and inferior quadrants being apparent while the nasal quadrant only showed a tendency to thin. The results of the present study are consistent with the findings of Barboni *et al*(18). The mean values of RNFL thickness in the nasal quadrant decreased at 10–12 months, which indicated that the RNFL in the nasal quadrant was thinning continuously. After 12 months, all measurements of RNFL showed significant thinning, and significant atrophy of RNFL in the nasal quadrant was observed.

Furthermore, no significant differences were identified in the BCVA between groups in the present study, and no linear correlation between the BCVA and RNFL thickness was observed. These results are consistent with the clinical observations of patients with LHON, since their visual acuity remained stable at the lowest level 6 months following disease onset (9,20). Certain patients even showed visual recovery to a certain degree while their RNFLs continued to shrink. In five patients with a disease duration of >20 years, the 360°-average RNFL thickness was relatively low, but not the lowest among all the study participants, indicating that the RNFL thickness varied in patients with LHON according to the time sequence recorded. A larger sample size may further elucidate this phenomenon.

In conclusion, the present study demonstrates the unique features of changes in RNFL thickness from the onset of LHON to 18 months and provided noteworthy information.

## Figures and Tables

**Table I:** Demographic information of the patients in each group.

Demographics	Group 1	Group 2	Group 3	Group 4	Group 5	Control
Gender						
Male	14	13	10	9	15	10
Female	0	1	0	1	5	5
Age, years (range)	15.4 (4–29)	19 (12–34)	21.5 (7–45)	17.7 (7–28)	28.9 (15–45)	24.9 (7–43)
Onset age, years (range)	15.1 (4–29)	18.8 (12–33)	20.7 (6–44)	17.1 (6–28)	17.6 (12–36)	-
ADV, months (range)	1.3 (0.3–3)	4.3 (3.3–5)	7.2 (6–9)	10.5 (9–12)	137.5 (14–360)	-
LogMAR BCVA score	1.5 (0.4–2.9)	1.6 (1.0–2.9)	1.6 (0.3–2.4)	1.6 (0.1–2.5)	1.8 (0.1–4.1)	-

ADV, average duration of disease; Logmar, logarithm of the minimal angle of resolution; BCVA, best corrected visual acuity; group 1, disease duration ≤3 months; group 2, disease duration 4–6 months; group 3, disease duration 7–9 months; group 4, disease duration 10–12 months; group 5, disease duration >12 months.

**Table II:** Mean values of the 360°-average RNFL thickness and the RNFL thickness in the temporal, superior, nasal and inferior quadrants in each group.

Group	360°-average (μm)	T (μm)	S (μm)	N (μm)	I (μm)
1	119.3±31.6	75.1±30.1	157.8±48.8	78.1±19.5	161.1±46.1
2	107.1±19.3	59.4±16.5	150.3±36.8	75.3±12.9	143.1±28.2
3	87.4±12.7	48.1±9.5	118.5±16.1	66.6±12.5	116.3±25.8
4	68.1±11.0	44.7±8.3	84.0±18.4	63.9±9.1	77.6±18.4
5	61.1±10.8	47.3±2.6	73.2±21.3	56.1±7.7	68.6±17.1
Control	100.5±8.7	76.1±16.0	130.2±16.2	65.7±10.2	130.3±14.0

RNFL, retinal nerve fiber layer; group 1, disease duration ≤3 months; group 2, disease duration 4–6 months; group 3, disease duration 7–9 months; group 4, disease duration 10–12 months; group 5, disease duration >12 months; T, temporal; S, superior; N, nasal; and I, inferior.
